# The Coordinated KNR6–AGAP–ARF1 Complex Modulates Vegetative and Reproductive Traits by Participating in Vesicle Trafficking in Maize

**DOI:** 10.3390/cells10102601

**Published:** 2021-09-30

**Authors:** Manfei Li, Ran Zhao, Yanfang Du, Xiaomeng Shen, Qiang Ning, Yunfu Li, Dan Liu, Qing Xiong, Zuxin Zhang

**Affiliations:** 1 College of Life Science, Yangtze University, Jingzhou 434025, China; mfli_maize@163.com; 2 National Key Laboratory of Crop Genetic Improvement, Hubei Hongshan Laboratory, Huazhong Agricultural University, Wuhan 430070, China; zhaoran@webmail.hzau.edu.cn (R.Z.); yanfangdu@webmail.hzau.edu.cn (Y.D.); shenxiaomeng@cau.edu.cn (X.S.); ningqiang_404@163.com (Q.N.); yunfuli@webmail.hzau.edu.cn (Y.L.); danliuHZAU@163.com (D.L.); xiongqing@webmail.hzau.edu.cn (Q.X.)

**Keywords:** ADP-ribosylation factor (Arf) GTPase, Arf GTPase-activating protein, inflorescence, vesicle transport, kernel number, Golgi apparatus

## Abstract

The *KERNEL NUMBER PER ROW6* (*KNR6*)-mediated phosphorylation of an adenosine diphosphate ribosylation factor (Arf) GTPase-activating protein (AGAP) forms a key regulatory module for the numbers of spikelets and kernels in the ear inflorescences of maize (*Zea mays* L.). However, the action mechanism of the KNR6–AGAP module remains poorly understood. Here, we characterized the AGAP-recruited complex and its roles in maize cellular physiology and agronomically important traits. AGAP and its two interacting Arf GTPase1 (ARF1) members preferentially localized to the Golgi apparatus. The loss-of-function *AGAP* mutant produced by CRISPR/Cas9 resulted in defective Golgi apparatus with thin and compact cisternae, together with delayed internalization and repressed vesicle agglomeration, leading to defective inflorescences and roots, and dwarfed plants with small leaves. The weak *agap* mutant was phenotypically similar to *knr6*, showing short ears with fewer kernels. AGAP interacted with KNR6, and a double mutant produced shorter inflorescence meristems and mature ears than the single *agap* and *knr6* mutants. We hypothesized that the coordinated KNR6–AGAP–ARF1 complex modulates vegetative and reproductive traits by participating in vesicle trafficking in maize. Our findings provide a novel mechanistic insight into the regulation of inflorescence development, and ear length and kernel number, in maize.

## 1. Introduction

Maize is a very important grain and feed crop with the highest yield in the world. The grain yield of maize (*Zea mays* L.) is closely correlated with the number of kernels produced from the ear inflorescence. During normal development, the female inflorescence meristem (IM) gives rise to indeterminate spikelet pair meristems (SPMs). Each SPM differentiates into two determinate spikelet meristems (SMs), and their development is terminated by the formation of floral meristems (FMs). The lower florets produced from FMs are abortive, whereas the fertile upper florets develop into kernels after double fertilization [1,2,3,4]. The phytohormone auxin plays a critical role in the development of inflorescences and florets in maize. The loss of functions of auxin biosynthesis-related genes, such as *SPARSE INFLORESCENCE1* [5] and *VANISHING TASSEL2* [6], results in greatly reduced numbers of spikelets and florets, shown as sparse or barren inflorescences. In addition to the genes involved in auxin synthesis, those involved in auxin localization and signaling participate in the regulation of axillary meristem (AM) development [7]. Polar auxin transport is mediated by the auxin influx carrier AUXIN/LIKE AUXIN proteins and the auxin efflux carrier PIN-FORMED proteins (PINs) [8,9]. In maize, *BARREN INFLORESCENCE2* (*BIF2*) encodes a PINOID serine/threonine kinase that phosphorylates ZmPIN1a, an ortholog of *Arabidopsis thaliana* PIN1 [10,11], and *BIF1* and *BIF4* encode two members of the AUXIN/INDOLE-ACETIC ACID protein family [7]. The mutation of any of these three genes results in barren inflorescences with fewer spikelets or kernels, indicating that the genes involved in polar auxin transport or auxin signaling play key roles in reproductive axillary meristem and lateral primordia initiation and development [7,11,12].

The polar localization of auxin carriers is established by the cell trafficking system [13,14,15,16]. Adenosine diphosphate-ribosylation (ADP-ribosylation) is a post-translational modification (PTM) for macromolecules, and its associated with DNA-damage repair, DNA replication, transcription, cell division, signal transduction, stress and infection responses, etc. [17,18]. ADP-ribosylation factor (Arf) GTPases (ARFs) are grouped into five subfamilies and are crucial factors involved in intracellular membrane trafficking [19]. The fungal toxin Brefeldin A (BFA) inhibits vesicle trafficking by affecting Arf guanine-nucleotide exchange factors (Arf-GEFs), which are required for the cycling of both GDP- and GTP-bound ARFs. Auxin efflux facilitators, such as PIN1, are regulated by GNOM, an Arf-GEF localized to Golgi cisternae, and act in trans-Golgi network/early endosome (TGN/EE) maintenance in Arabidopsis [20,21,22]. The localization of the auxin influx facilitator AUX1 is regulated by Arf GTPase-activating protein (AGAP) through vesicle trafficking in rice [16]. When Arf-GEF is inhibited by BFA, PIN1s accumulate in the BFA compartments [21]. Additionally, AGAP contributes to the hydrolysis of ARF-bound GTP, which is the opposite reaction to the hydrolysis catalyzed by Arf-GEF. Additionally, AGAP domain (AGD) protein1 (AGD1), *vascular network defective3* (VAN3)/AGD3, NEVERSHED (NEV)/AGD5, AGD7, RPA/AGD10, and OsAGAP function in vesicle trafficking, with important implications in hormone signaling, polarized cell growth, and organ separation [16,23,24,25,26,27,28]. Therefore, AGAP–ARF complexes are considered molecular switches for polar auxin transport mediated by the intracellular trafficking system. 

Plant ARFs are targeted to various subcellular compartments, including the Golgi apparatus, post-Golgi organelles, and plasma membrane [29,30,31], and co-localize with AGAPs. In addition, AGAPs physically interact with ARF1 members, including VAN3/AGD3, RPA/AGD10, and AGD7 [23,25,27], inferring that AGAPs are distributed on distinct membranes to specifically activate different ARFs. ARF1 subfamily proteins are the best studied plant small GTPases and are involved in several trafficking routes, including protein trafficking at the Golgi apparatus/endoplasmic reticulum (ER) interface [29,30,32,33], and the transport of vacuolar proteins [34]. 

Members of the AGAP family share a common N-terminal GAP domain bearing a Cys4 zinc-finger motif [35]. This domain activates the GTPase activity of ARF by interacting with ARF effector domains; whereas the C-terminal regions of AGAPs contain conserved pleckstrin homology, ankyrin repeats, or Ca^2+^-binding motifs [36,37]. A maize AGAP interacts with KERNEL NUMBER PER ROW6 (KNR6), a serine/threonine protein kinase in vitro, to regulate the number of kernels on mature ears [38]. To further understand the mechanism of the KNR6–AGAP module in the regulation of the ear inflorescence and kernel number, independent CRISPR/Cas9 lines of *AGAP* and *KNR6* were created, and a genetic interaction between them was revealed, as were their roles in cellular physiology. We hypothesize that the KNR6–AGAP complex regulates kernel number and ear length by participating in vesicle trafficking during endocytosis through interactions with two ARF1 subfamily proteins in maize.

## 2. Materials and Methods

### 2.1. Sequence Extraction and Phylogenetic Analysis

To predict ARF proteins encoded in the maize genome, the raw ARF hidden Markov model (HMM) file, downloaded from the EMBL-EMI protein database, was used to query the B73 genomic database (Zea_mays.AGPv4.pep.all.fa; http://plants.ensembl.org/, accessed on 2020) using the HMM, then those predicted hits were aligned by ClustalW2 to produce a set of high-quality proteins with E-value < 1 × 10^−20^ and an intact GTPase domain. The high-quality protein set was used to construct a maize-specific ARF HMM using hmmbuild in the HMMER v3 suite [39]. The maize-specific ARF HMM was used to query the B73 genomic database again, and the hits with E-value < 0.01 were referred to as maize ARF proteins. The 16 sequences were aligned using MUSCLE 3.8.31 (http://www.drive5.com/muscle/downloads.htm, accessed on 2020), and the phylogenetic trees were constructed using MEGA-X 10.1 with the maximum-likelihood method.

### 2.2. Vector Construction and Genetic Transformation

Two guide RNAs (gRNAs), GGCAAUAAGAUUGGUGAGGG and GCUGUGUAGAAGGAAACCAC (Figure S1), that target the coding region of *KNR6* were designed using CRISPR-P 2.0 (http://crispr.hzau.edu.cn/CRISPR2/, accessed on 2017). Similarly, the gRNAs GACGGAUUUGAGGCCCAACA and GUGGCUCUCCAGAUCCAAAA, targeting the *AGAP* gene, were also designed (Figure S2). These gRNAs were then synthesized by the GeneCreate Company (Wuhan, China). The synthesized gRNAs were transformed independently into ZmUbi–hspCas9 through a recombination reaction using CloneExpress Multis (Vazyme Biotech, Nanjing, China). The *KNR6*–ZmUbi–hspCas9 and the *AGAP*–ZmUbi–hspCas9 constructs were transformed into *Agrobacterium tumefaciens* strain EHA101 and then were introduced independently into immature embryos of maize-inbred line KN5585 through Agrobacterium-mediated transformations [38] by the Wimibio Company (Changzhou, China). Independent T_0_ transgenic plants were identified by PCR genotyping of the *Bar* gene, and the gene-specific editing was identified using gene-specific PCR and sequencing. The PCR primers used are listed in Table S1. *KNR6*-edited lines, *knr6^cr1^* and its respective non-transgenic line, *KNR6^NT1^*, as well as two *AGAP*-edited lines, *agap^cr1^* and *agap^cr2^*, and their respective non-transgenic lines, *AGAP^NT1^* and *AGAP^NT2^*, were developed.

### 2.3. Plant Materials and Phenotypic Identification

All of the gene-edited lines and their respective non-transgenic lines were phenotyped during spring 2020 in Wuhan (30° N, 114° E), China; *knr6^cr1^* was crossed to *agap^cr1^* and self-crossed during winter 2019 in Sanya (18.34° N, 109.62° E), China. Wild-type, two single-mutant and double-mutant individuals were selected from the segregating F_2_ population of 607 plants by genotyping *KNR6* and *AGAP*. Individuals of the four genotypes were phenotyped during spring 2020 in Wuhan. The inflorescence meristem lengths (μm) were measured under a scanning electron microscope. Botanical characteristics, including plant height (cm), ear height (cm), and tassel length (cm), were measured during the adult period. The ear length (cm), kernel number per row (KNR), and kernel number per ear were determined on air-dried ears.

### 2.4. Luciferase (Luc) Complementation Image Assay

The full-length coding sequences (CDSs) of AGAP and the *ARF1* family genes were cloned into 35S::CLuc and 35S::NLuc independently using the recombinant enzyme combinations *Kpn*I/*Sal*I and *BamH*I/*Sal*I, respectively. The constructs were introduced into *A. tumefaciens* strain GV3101 (Weidibo, Shanghai, China), and resulting strains harboring ARF1s-NLuc and AGAP-CLuc were coinfiltrated into *Nicotiana benthamiana* leaves. After culturing in the lysogeny broth medium supplemented with 100 µg mL^−1^ Kanamycin and 50 µg mL^−1^ Rifampicin until the optical density measured at 600 nm reached 0.6, the Agrobacterium pellet was collected and homogenized in suspension solution (10 mM MgCl_2_, 10 mM 4-Morpholineethanesulfonic acid (MES) at pH = 5.6, and 100 µM acetosyringone) for 2 h in the dark. The *N. benthamiana* plants were grown under greenhouse conditions with a 14 h/10 h light/dark photocycle at 23 °C. Leaves of *N. benthamiana* (5 to 6 weeks old) were inoculated by filtration through a 1 mL syringe with the *A. tumefaciens* strain carrying the appropriate plasmid construct [40]. Two days after inoculation, 1 mM luciferin was sprayed onto the inoculated leaves. The sprayed leaves were then maintained in the dark for 6 min to quench the fluorescence. A low-light cooled CCD imaging apparatus (Carestream Health, Rochester, NY, USA) was used to capture the luciferase image.

### 2.5. Yeast Three-Hybrid (Y3H) Assay

The full-length CDSs of *Zm00001d043113* (*ARF1.1*) and *Zm00001d008295* (*ARF1.2*) was cloned into pGADT7 using *EcoR*I. The full length CDS of KNR6 was cloned into pBridge (pBridge-KNR6) using *EcoR*I, and then the full-length CDS of *AGAP* were cloned into pBridge-KNR6 using *Bgl*II. The constructs of pGADT7-ARF1s and pBridge-KNR6-AGAP were transformed into yeast strain Y2H and spread onto plates containing SD/-Met/-Trp. To investigate if the AGAP can participate in the interaction as a bridge, the transformation mixture was serially diluted (10^−1^, 10^−2^, 10^−3^, 10^−4^) and grown on SD/-Met/-Trp and SD/-His/-Leu/-Met/-Trp plates; pGADT7-ARF1.1 and pBridge-KNR6 were used as negative control.

### 2.6. Subcellular Localization

The full-length CDSs of *AGAP*, *ARF1.1,* and *ARF1.2* were cloned into pS1304-mCherry using *Spe*I digestion. The Golgi maker ST (*AT2G03760*) in *Arabidopsis thaliana* was cloned into pM999-GFP using *Xba*I. Plasmids were purified using NucleoBond Xtra Midi (Macherey Nagel, Berlin, Germany). The leaf tissues of 10- to 12-day-old maize line B73 grown in a dark incubator at 28 °C were cut into small squares (5 to 10 mm^2^) with a new razor blade and incubated with 50 mL of enzyme solution (0.25% Macerozyme (Yakult Honsha Co., Ltd., Tokyo, Japan) R-10, 1.0% Cellulase (Yakult Honsha Co., Ltd.) R-10, 400 mM mannitol, 8 mM CaCl_2_, and 5 mM Mes-KOH, pH 5.6). The enzyme solution with leaf tissues was evacuated at ~30 kPa for 20 min and then with gentle agitation (30 to 50 rpm) for 5 h at 25 °C. After incubation, the protoplast suspension was filtered through 100 μm mesh and protoplasts were collected by centrifugation at 100 g for 2 min. The pelleted protoplasts were resuspended in 5 to 10 mL of W5 solution (154 mM NaCl, 125 mM CaCl_2_, 5 mM KCl, 5 mM glucose, and 1.5 mM Mes-KOH, pH 5.6), and centrifuged for 2 min at 100 g. The intact protoplasts at the interface were transferred to a new Falcon tube containing 20 mL of W5 solution. The protoplasts were pelleted again by centrifugation at 100 g for 2 min and resuspended in 20 mL of W5 solution. The protoplasts were incubated on ice for 30 min. The protoplasts were pelleted again at 100 g for 2 min and resuspended in MMG solution (400 mM mannitol, 15 mM MgCl_2_, and 5 mM Mes-KOH, pH 5.6) at a density of 5 × 10^6^ protoplasts/mL.

To transform DNA into protoplasts, plasmid DNA (10 to 20 μg total at a concentration of 2 mg/mL) was added to 100 μL of protoplast suspension followed by 110 μL of PEG solution (400 mM mannitol, 100 mM Ca(NO_3_)_2_, and 40% polyethylene glycol 4000). The mixture was mixed gently and incubated for 15 min at room temperature. After incubation, the mixture was diluted with 440 μL of W5 solution. Protoplasts were recovered by centrifugation at 100 g for 2 min, resuspended in 1 mL of WI solution (500 mM mannitol, 20 mM KCl, and 5 mM Mes-KOH, pH 5.6), and incubated at 25 °C in the dark for 12–16 h. Expression of protein was monitored after transformation. Fluorescent signals were visualized using a FV1200 laser scanning confocal fluorescence microscope (Olympus, Tokyo, Japan) with the 488 nm laser line for GFP and 552 nm laser line for mCherry. Data were then processed using Radial Profile Plot in ImageJ 1.53j (Wayne Rasband and contributors, National Institutes of Health, USA) 

### 2.7. Transmission Electron Microscopic Observation of Golgi Apparatus

For transmission electron microscopy, 2–3 cm seedling roots were cut into 1–2 mm segments and fixed in 4% glutaraldehyde in 0.05 M sodium phosphate buffer overnight at 4 °C. After fixation, samples were stained with 1% osmium tetroxide, dehydrated through an ethanol series, and embedded in Spurr’s resin (London Resin Company, London, UK). Sections (60–70 nm) were cut with a diamond knife, mounted on copper mesh grids, and stained with 4% uranyl acetate followed by Reynolds lead citrate. Sections were then examined using an H-7650 transmission electron microscope (Hitachi, Tokyo, Japan) at 100 kV.

### 2.8. FM4-64 Internalization Assay

The FM4-64 internalization assay was carried out as described by Fan et al. (2013) [41] and Wang et al. (2020) [42]. The *agap^cr^* and *AGAP^NT^* seedlings were incubated in 5 μM FM4-64 (Invitrogen, Carlsbad, CA, USA) for 10 min at room temperature. Then, the root cells that had been dyed with FM4-64 were transferred into 10 μM BFA. The roots were hand-sectioned, and the FM4-64 internalization was monitored using a FV1200 laser scanning confocal fluorescence microscope (Olympus) with a 552 nm laser line. The number of FM4-64 labeled puncta internalized per cell and the BFA bodies’ size were determined using ImageJ 1.51K.

## 3. Results

### 3.1. AGAP Gene Shows Pleiotropy in Vegetative and Reproductive Traits

**Figure 1:**
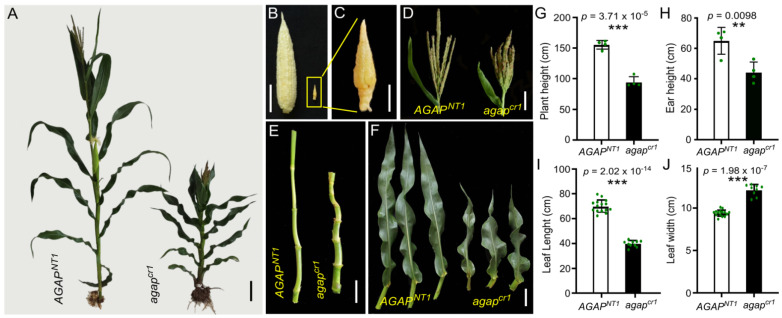
Loss of *AGAP* function significantly alters vegetative and reproductive traits. (**A**) A semi-dwarf plant generated from the gene knockout line (right, *agap^cr1^*) compared with its non-transgenic sibling (left, *AGAP^NT1^*). (**B**–**D**) Loss of *AGAP* function alters the architecture of inflorescences. In the gene knockout line *agap^cr1^* (right), ear growth was obviously inhibited (**B**,**C**), and the tassel was claw-like (**D**). (**E**,**F**) The gene knockout *agap^cr1^* plant (right) had shorter and compact internodes (**E**) and a reduced leaf size (**F**) compared with its non-transgenic sibling (left, *AGAP^NT1^*). (**G**–**J**) Measured phenotypic characteristics of agronomically important traits: Plant height (**G**), ear height (**H**), leaf length (**I**) and leaf width (**J**) were significantly different between *AGAP^NT1^* and *agap^cr1^.* Phenotypes were assessed at Wuhan, China, in spring 2020. The values in (**G**–**J**) are the means ± s.d.s, and the significance levels of differences were estimated using a one-way ANOVA. Scale bars = 10 cm in (**A**), 5 cm in (**B**,**D**–**F**), and 1 cm in (**C**); ** indicates a statistical difference at the *p* < 0.01 level, *** indicates a statistical difference at the *p* < 0.001 level.

A maize AGAP is phosphorylated by KNR6 to modulate the length of inflorescence meristems and, in turn, the lengths of mature ears and number of kernels borne per ear [38]. The studied AGAP contains 385 amino acid (aa) residuals with a conserved Arf_GAP domain (AGD) from the 50th to 164th aa and a C2 domain, having five Ca^2+^ binding pockets, from the 226th to 372nd aa (Figure S3A). It is highly homologous with SORBI_3009G121200 in *Sorghum bicolor*, SEVIR_3G267200v2 in *Setaria viridis*, and AT3G07940 in *A. thaliana* (Figure S3B,C). To further reveal the roles of the KNR6–AGAP module in the ear inflorescence, two loss-of-function *agap* mutants (*agap^cr1^* and *agap^cr2^*) were created using CRISPR/Cas9; *agap^cr1^* has a 450-bp deletion between targets 1 and 2, and *agap^cr2^* has a 1-bp deletion near the protospacer-adjacent motif of target 1. The deduced proteins translated from these two types of edited *AGAP* may lose both the AGD and the C2 domains to produce truncated proteins (Figure S2A,B). Phenotypic observations revealed dramatic changes between the *agap^cr1^* and its wild-type sibling (*AGAP^NT1^*). Specifically, *agap^cr1^* produced a shorter plant (94.45 ± 8.76 cm, *n* = 4) and smaller ear (44.28 ± 6.75 cm) than *AGAP^NT1^* (155.50 ± 7.12 cm and 65 ± 8.83 cm, respectively), and compact and asymmetrical internodes (Figure 1A,E,G,H). The growth of the *agap^cr1^* ear inflorescence was strongly suppressed, resulting in stunted ears (Figure 1B,C). The *agap^cr1^* tassel was claw-like, although the tassel length difference between *agap^cr1^* (22.25 ± 3.72 cm) and wild-type (27.15 ± 2.83 cm) plants was not statistically significant (*p* = 0.08; Figure 1D). The *agap^cr1^* leaves, at 39.86 ± 2.61 cm long and 12.13 ± 0.66 cm wide, were shorter but wider than wild-type leaves (69.82 ± 5.34 cm long and 9.41 ± 0.37 cm wide, Figure 1F,I,J). In the *agap^cr2^* plants, the ear inflorescence and ear traits showed slight but statistically significant changes, with reduced ear lengths and kernel numbers, whereas the agronomically important traits, including plant and leaf architecture, did not significantly differ between *agap^cr2^* and its non-transgenic sibling (*AGAP^NT2^*) (Figure S4A–K). Thus, the loss of both AGD and C2 domains in AGAP produced strong effects on vegetative and reproductive traits. Although both mutants were predicted to translate a truncated and non-functional AGAP, the phenotype of the *agap^cr1^* was quite different from that of the *agap^cr2^*. 

### 3.2. AGAP Genetically Interacts with KNR6

**Figure 2:**
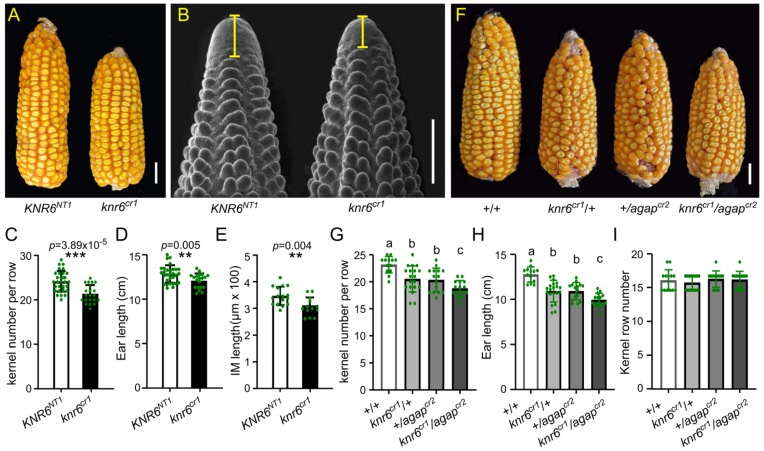
AGAP genetically interacts with KNR6 to affect ear length and kernel number per row. (**A**,**B**) *knr6^cr1^* produced shorter mature ears (**A**) and ear inflorescence meristems (**B**) relative to its non-transgenic sibling (*KNR6^NT1^*). (**C**–**E**) The ear inflorescence meristem length (**D**), mature ear length (**E**), and kernel number per row (**F**) of the *KNR6* knock-out line (*knr6^cr1^*) were significantly different from those of its non-transgenic sibling (*KNR6^NT1^*). (**F**) Single-gene mutants (*knr6^cr1^*/+ and +/*agap^cr2^*) and the double-gene mutant (*knr6^cr1^*/*agap^cr2^*) showed smaller ears than wild type. (**G**–**I**) The double-gene mutation enhanced the phenotypic effects of the single-gene mutants. Phenotypic differences between mutants and wild type were revealed for kernel number per row (**G**), ear length (**H**), and kernel row number (**I**). Data are shown as the means ± s.d.s. The significances of the differences at *p* < 0.05 were determined using the Tukey HSD test. Scale bars = 2 cm in (**A**,**F**) and 200 μm in (**B**); ** indicates a statistical difference at the *p* < 0.01 level, *** indicates a statistical difference at the *p* < 0.001 level.

AGAP physically interacts with KNR6 [38]. To determine the interaction in vivo, we created a *KNR6* knock-out mutant (*knr6^cr1^*) in which a 261-bp-encoding region between the second and third exons was deleted (Figure S1). The ear inflorescences and mature ears of the *knr6^cr1^* showed reduced lengths, along with fewer kernels, relative to those of its non-transgenic sibling (Figure 2A–E). These phenotypes were similar to those of RNAi families reported by Jia et al. (2020) [38], indicating the key roles of *KNR6* in the development of ear inflorescences and kernels. Next, the *knr6^cr1^* mutant was crossed to the weak *agap^cr2^* mutant to develop an F_2_ segregation population. In this population, we identified the four genotypes, double-mutant *knr6^cr1^*/*agap^cr2^*, two single mutants, *knr6^cr1^*/*+* and *+*/*agap^cr2^*, and the +/+ wild type, by genotyping. Compared with the wild-type individuals, each of the single mutants showed shorter ears with fewer kernels (Figure 2F,G–I). Importantly, compared with the average ear lengths of 10.91 ± 1.19 cm and 10.91 ± 0.95 cm in the single mutants *knr6^cr1^*/*+* and *+*/*agap^cr2^*, respectively, the average ear length of the double mutant (*knr6^cr1^*/*agap^cr2^*) was 9.93 ± 0.76 cm (*n* = 14), indicating that double mutants had shorter ears than each of the single mutants (Figure 2H). Similarly, the average kernel number per row (KNR) in the double mutant was 18.73 ± 1.49 (*n* = 13), which was fewer than in the single mutants *knr6^cr1^*/*+* (20.53 ± 2.41, *p* = 0.0124) and *+*/*agap^cr2^* (20.33 ± 2.19, *p* = 0.0276) (Figure 2G). Thus, mutations at both genes enhanced the defective phenotypes resulting from the single gene mutation, suggesting that *AGAP* genetically interacts with *KNR6* to influence ear length and KNR.

### 3.3. AGAP Participates in Vesicle Trafficking

**Figure 3:**
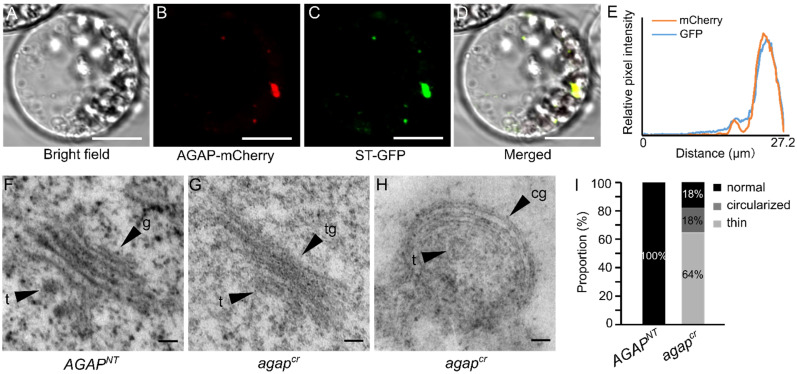
AGAP protein localized on the Golgi apparatus and alters structure of a partial Golgi apparatus. (**A**–**E**) Subcellular localization of AGAP and Golgi maker: (**A**) bright field, (**B**) mCherry for AGAP localization, (**C**) is the marker for Golgi, (**D**,**E**) shows the overlap of AGAP and Golgi maker. Scale bar = 10 μm. (**F**) Normal Golgi apparatus morphology in *AGAP^NT^* cells. (**G**,**H**) Thin (**G**) and curved (**H**) Golgi apparatus were observed using a transmission electron microscope. (**I**) Proportions of different Golgi types in *AGAP^NT^* and *agap^cr^*. Scale bar = 100 nm; tg, thin Golgi apparatus; g, Golgi cisternae; t, trans-Golgi network; cg, curved Golgi apparatus.

To determine the roles of AGAP, we first examined the transient expression of AGAP–GFP in tobacco (*Nicotiana benthamiana* L.) leaves. We found that AGAP–mCherry signals were enriched in the cytoplasm (Figure 3A,B). Furthermore, to understand where the organelles AGAP–mCherry localized, *Arabidopsis thaliana* ST-GFP was used as a marker to label the Golgi compartments. The AGAP–mCherry signal overlapped the signals of Golgi marker, with the similar relative pixel intensity (Figure 3C–E), inferring that AGAP localized on membranes of the Golgi apparatus. Moreover, through transmission electron microscope observations, we found that 72% of the Golgi apparatus (*n* = 11) in the *agap^cr^* cells exhibited abnormal architectural features, with thin (64%) or circularized (18%) structures, and compact Golgi cisternae (Figure 3F–I), instead of the typically flat and linear Golgi cisternae in *AGAP^NT^* cells (Figure 3F). The circularized Golgi apparatus had curved Golgi cisternae, and the TGN/EE was maintained in the Golgi cisternae (Figure 3H). The results indicate that AGAP is required for Golgi organization.

**Figure 4:**
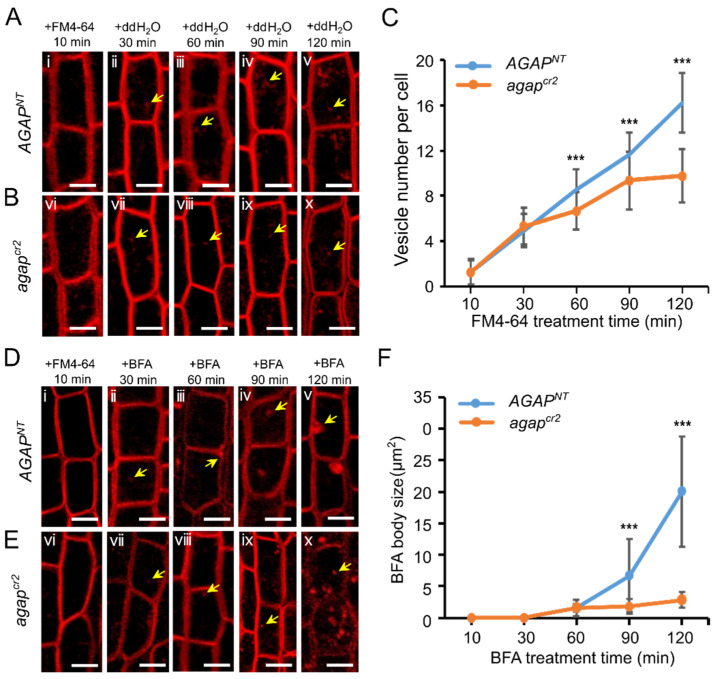
The numbers and agglomeration of vesicles in *AGAP^NT^* and *agap^cr2^* cells. (**A**,**B**) The FM4-64-labeled vesicles in *AGAP^NT^* and *agap^cr2^* cells after 10 (i and vi, respectively), 30 (ii and vii, respectively), 60 (iii and viii, respectively), 90 (iv and ix, respectively), and 120 min (v and x, respectively) of FM4-64 staining. Arrowheads indicate FM4-64-labeled vesicles. (**C**) To test for FM4-64 treatment effect on the response variables with time, repeated measures ANOVA was performed and time as the within-subject factor (general linear model (GLM) in SPSS 16.0). (**D**,**E**) Vesicle agglomeration in *AGAP^NT^* and the *agap^cr2^* cells. The fungal toxin Brefeldin A (BFA) bodies were revealed by BFA re-treatment in the FM4-64-labeled cells after 10 (i and vi, respectively), 30 (ii and vii, respectively), 60 (iii and viii, respectively), 90 (iv and ix, respectively), and 120 (v and x, respectively) min BFA treatments. (**F**) To test for FM4-64 and BFA treatment effect on the response variables with time, repeated measures ANOVA was performed the same as in FM4-64 treatment. Scale bars in (**A**,**B**,**D**,**E**) = 20 μm; *** indicates a statistical difference at the *p* < 0.001 level.

Because the roles of the Golgi apparatus are to process, sort and transport proteins synthesized by the ER and then send them to specific target membranes or secrete them out of cells, we observed the organelle compartments in the cells of 2–3 cm roots of 3-day-old seedlings using FM4-64 and BFA treatments. The vesicles in cells were identified by FM4-64-labeled fluorescent puntca, and the average vesicle number per cell was counted every 30 min after the FM4-64 treatment. A significant difference in the vesicle number per cell was observed between wild-type and *agap^cr2^* cells at 60 min after the FM4-64 treatment. The vesicle number per cell in *agap^cr2^* cells was 6.704 ± 1.64 (*n* = 27), less than that in *AGAP^NT^* cells (8.556 ± 1.62, *n* = 27; F = 18.729, *p* < 0.001), at 60 min after the treatment. The average vesicle numbers slowly increased in *agap^cr2^* cells during the treatment process, reaching 9.333 ± 2.61 (*n* = 27) and 9.704 ± 2.46 (*n* = 27) at 90 and 120 min, respectively, after treatment. However, the vesicle number and rate of increase in *AGAP^NT^* cells were much greater than in *agap^cr2^* cells (F = 18.433, *p* < 0.001 at 90 min and F = 95.061, *p* < 0.001 at 120 min after treatment) (Figure 4A–C), indicating that the internalization of FM4-64-labeled puntca in the *agap^cr2^* cells was delayed compared with in *AGAP^NT^* cells.

The fungal toxin BFA drives the rapid agglomeration of endomembrane compartments and the accumulation of membrane proteins into cellular structures, termed ”BFA bodies” [43,44,45]. Therefore, we re-treated those FM4-64-treated cells with BFA and observed the states and sizes of BFA bodies in the root epidermal cells.

We found that after 60 min BFA treatment, the small dots indicating FM4-64-labeled transport vesicles (Figure 4D) were combined into patches (BFA bodies). The average sizes of BFA bodies in *AGAP^NT^* cells (1.601 ± 0.69 μm^2^) and *agap^cr2^* cells (1.047 ± 0.97 μm^2^) have statistical significance (F = 6.393, *p* = 0.018). The *AGAP^NT^* cells have average sizes of 6.173 ± 6.02 μm^2^ (*n* = 26) and 19.932 ± 9.22 μm^2^ (*n* = 26) at 90 and 120 min, respectively, after the BFA treatment (Figure 4D). However, in the *agap^cr2^* cells, the sizes of the BFA bodies were only 1.791 ± 1.23 μm^2^ (*n* = 26) and 2.814 ± 1.28 μm^2^ (*n* = 26) at 90 and 120 min, respectively, after the BFA treatment (Figure 4E,F), which were significantly smaller than the sizes in wild-type cells at the same respective time-points (F = 21.364, *p* < 0.001 and F = 111.408, *p* < 0.001, respectively). The results indicate that AGAP is required for vesicle agglomeration. 

### 3.4. AGAP Physically Interacts with Two Members of the Arf GTPase 1 (ARF1) Protein Subfamily

**Figure 5:**
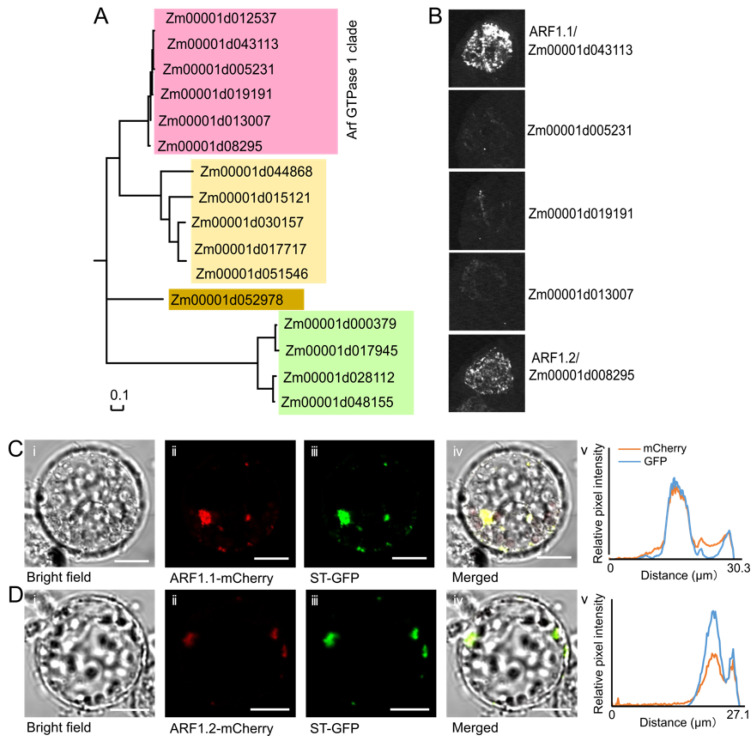
AGAP interacts with two ARF1 members on the Golgi apparatus. (**A**) In total, 16 putative maize ARF1s were grouped into 4 clades. (**B**) AGAP physically interacted with ARF1.1 and ARF1.2. The interactions were assessed using luciferase complementation image assays (**B**). (**C**) Subcellular localization of ARF1.1 and Golgi maker. (**C**-i) Bright field. (**C**-ii) mCherry for ARF1.1 localization. (**C**-iii) is the marker for Golgi. (**C**-iv) and (**C**-v) show the overlap of ARF1.1 and Golgi maker. Scale bar = 10 μm. (**D**) Subcellular localization of ARF1.2 and Golgi maker. (**D**-i) Bright field. (**D**-ii) mCherry for ARF1.2 localization. (**D**-iii) is the marker for Golgi. (**D**-iv) and (**D**-v) shows the overlap of ARF1.2 and Golgi maker. Scale bar = 10 μm.

The AGAPs act by binding to GTPases and contribute to the hydrolysis of GTP-bound ARFs [46,47]. In the maize B73 genome, 16 ZmARFs were annotated and divided into four clades (Figure 5A). To identify the ZmARFs interacting with the studied AGAP, we isolated five out of six annotated members of the ZmARF1 subfamily by PCR and analyzed their interactions with the AGAP using luciferase complementation image assays. We found only two proteins, Zm00001d043113 (ARF1.1) and Zm00001d008295 (ARF1.2), which interacted with the AGAP (Figure 5B), whereas the remaining three members did not show AGAP-interacting signals, indicating that the AGAP proteins preferentially bind to ARF1.1 and ARF1.2. Additionally, the GTP-bound active form of ARF is recruited to the Golgi compartments [48]. Consequently, the subcellular localization showed that ARF1.1 and ARF1.2 predominantly localized on the Golgi apparatus (Figure 5C,D). Bioinformatics predictions revealed that neither of the ZmARF1 proteins, nor the AGAP, have transmembrane domains; therefore, we hypothesize that these two ZmARF1 proteins and the AGAP protein may be recruited to the Golgi apparatus by Golgi-bound effectors. 

## 4. Discussion

The ear inflorescence developmentally originates from the apical meristem of axillary branches after the reproductive transition. The ear inflorescence meristem successively differentiates into indeterminate SPMs, determinate spikelet meristems, and then terminates with FM production [49]. The ear inflorescence meristem axially generates a variable number of florets and, in turn, kernels, which are developed from florets after double fertilization. Therefore, the number of kernels arranged axially on the ear is agronomically referred to as KNR. Undoubtedly, KNR is closely correlated with the determinacy of SPMs during ear inflorescence development. A complex functional hierarchy of genes participates in the regulation of SPM determinacy in maize, including genes involved in the CLAVATA–WUSCHEL (CLV–WUS) negative feedback loop, phytohormone biosynthesis and signaling, and in microRNA-mediated post-transcriptional regulation [12,50,51,52]. A recent study found that *KNR6* is a pleiotropic quantitative trait locus for ear length and KNR, and its underlying gene encodes a serine/threonine protein kinase that phosphorylates an AGAP that controls GTP-binding protein activity levels [38]. Because of the phosphorylation roles of serine/threonine protein kinases on small GTPase-coupled receptors [36,53] and maize AGAP [38], and the functions of AGAP in the transport of auxin in rice and Arabidopsis [16,45,54,55], it has been proposed that KNR6 acts in auxin-dependent inflorescence development by mediating AGAP phosphorylation [38]. However, downstream pathways and biological processes regulated by phosphorylated AGAP, which will expand our knowledge of the molecular regulation of reproductive axillary meristem determinacy, are relatively unknown in maize. 

The AGAP family of genes is involved in plant organ differentiation and formation [16,23,25]. In Arabidopsis, an AGAP, VAN3/AGD3 controls vein patterning by participating in the *trans*-Golgi network [23]. A class II AGAP protein, Arabidopsis RPA/AGD10, modulates root hair development by activating the ARF1 subfamily of proteins [25]. In rice, OsAGAP negatively controls root growth and development by modulating the auxin influx pathway [16]. In this study, we found that a weak mutant of maize *AGAP* alters ear inflorescence-related traits, producing shorter inflorescence meristem and ear length, together with fewer florets and kernels. The strong mutant of maize *AGAP* showed pleiotropic effects on vegetative and reproductive traits, producing dwarfed plants, asymmetric internodes, and dramatically repressed ear development. We speculated that the 1-bp deletion (*agap^cr2^*) at the DNA level may be partially repaired or bypassed during translation to encode a functional AGAP protein, resulting in a weak mutant phenotype, whereas the large deletion at the DNA level (*agap^cr1^*) is not significantly repaired, leading to a strong mutant phenotype. The genetic effects of the *agap* mutant on the ear traits were enhanced by the *knr6* mutant, providing in vivo evidence for KNR6–AGAP protein interactions. These results demonstrate that the KNR6–AGAP complex is required for vegetative and reproductive development in maize. 

Generally, AGAPs, such as AGD7 and NEV/AGD5 in Arabidopsis, localize on membranes of the Golgi apparatus, and they participate in membrane trafficking from the Golgi apparatus to ER [27,56]. As in several known *AGAP* mutants [28,57], the Golgi apparatus showed a defective structure, the internalization of FM4-64 labeled puntca was delayed, and the agglomeration of the typical BFA bodies was not observed in the *agap* cells, indicating that the involvement of AGAP is required for intact Golgi structures and functions in vesicle trafficking. In addition to AGAP, GTPases are other key factors involved in endocytosis and the Golgi apparatus-to-ER retrograde trafficking in plants [27,58,59,60]. The Arf_GAP domain in AGAP proteins binds to ARFs and then catalyzes active GTP-bound ARFs into inactive GDP-bound ARFs, whereas GDP-bound ARFs are activated by Arf-GEF [60,61]. In Arabidopsis, VAN3/AGD3, AGD7, and RPA/AGD10 bind to ARF1 and then regulate ARF1 activity [23,25,27]. AGD8 and AGD9 also recruit GDP-bound ARF1 to Golgi apparatus [57]. We found that two maize ARF1 subfamily proteins physically interact with AGAP. In these cases, AGAP functions may be achieved by interactions with ARF1 proteins. Thus, the signaling mediated by the two ARF1 proteins requires the involvement of AGAP in maize. We also used the pBridge vector system to find that KNR6, AGAP, and ZmARFs could not form a ternary protein complex (Figure S5), although the protein–protein interactions were detected between KNR6 and AGAP, AGAP, and ZmARFs. 

**Figure 6:**
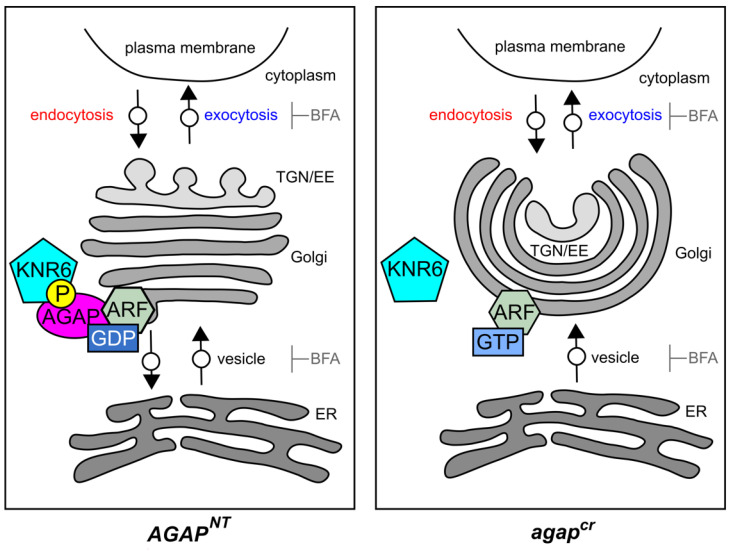
A potential working model of the KNR6–AGAP–ARF1 complex in maize. Under normal conditions, KNR6 binds to and phosphorylates AGAP^NT^, and the phosphorylated AGAP interacts with ARF1, leading to the release of vesicles from membranes. When treated with BFA, vesicles from exocytosis and ER-to-Golgi apparatus trafficking are disrupted, with the former gathering into TGN/EE and the latter forming BFA bodies. In *agap^cr^* cells, endocytosis and Golgi apparatus-to-ER retrograde trafficking involving AGAP are deficient. The vesicle efflux from TGN/EE is greater during exocytosis than the protein influx into TGN/EE during endocytosis, leading to the smaller TGN/EE. The number of vesicles transported from the ER to Golgi apparatus is greater than the number of vesicles transported from the Golgi apparatus to ER, leading to larger Golgi cisternae. When treated with BFA, the BFA bodies that originate from endocytosis and Golgi apparatus-to-ER trafficking are inhibited; therefore, the agglomeration of BFA bodies is delayed compared with in *AGAP^NT^* cells.

Using our results, we established a model of the KNR6–AGAP–ARF1 complex-mediated regulation of vegetative and reproductive traits in maize through its participation in vesicle trafficking (Figure 6). We propose that the serine/threonine-protein kinase KNR6 binds to and then phosphorylates the AGAP, and the phosphorylated AGAP interacts with two specific ARF1 proteins to form the KNR6–AGAP–ARF1 complex. The multi-factor complex is recruited by a specific set of effectors on the Golgi apparatus to hydrolyze GTP into GDP, while Arf-GEF catalyzes the displacement of pre-bound GDP, allowing its replacement with GTP [60,61]. The GTP-bound ARF1 is active, whereas the GDP-bound ARF1 is inactive. The inactive ARF1 is released from membranes after altering its conformation [62,63]. The small GTPase cycle is widely considered a switch mechanism in membrane trafficking [64]. In the *agap^cr1^* cells, GTP-bound ARF1 cannot be released from the Golgi apparatus because the GTP–GDP nucleotide cycle is interrupted, which further represses the internalization and agglomeration of vesicles and damages Golgi compartments. Thus, the failure of the ARF1 cycle interrupts the organization of vesicles from the Golgi apparatus and, in turn, causes cytological, physiological, and/or developmental malfunction through a set of unknown pathways. A proposed downstream pathway is an auxin-mediated regulatory mechanism, which remains to be further investigated.

## Data Availability

Not applicable.

## Supplementary Materials

The following are available online at https://www.mdpi.com/article/10.3390/cells10102601/s1. Table S1: Primers used in this study, Figure S1: The guide RNAs of KNR6 and editing types in KNR6 knockout lines, Figure S2: Guide RNAs of AGAP and editing types in AGAP knockout lines, Figure S3: Protein structure, phylogenetic tree, and conserved domain analyses of AGAP, Figure S4: Vegetative and reproductive traits of *AGAP^NT2^* and *agap^cr2^*, Figure S5: Two maize ARF1 proteins, KNR6 and AGAP could not form a ternary protein complex.
