# Supplementary material for: The Coordinated KNR6–AGAP–ARF1 Complex Modulates Vegetative and Reproductive Traits by Participating in Vesicle Trafficking in Maize
Source: Cells. 2021 Sep 30;10(10):2601. doi: 10.3390/cells10102601 (PMC8533723; doi:10.3390/cells10102601)
Supplement: Supplementary file 1 [file cells-10-02601-s001.zip › cells-1400938-supplementary-figures.pdf]

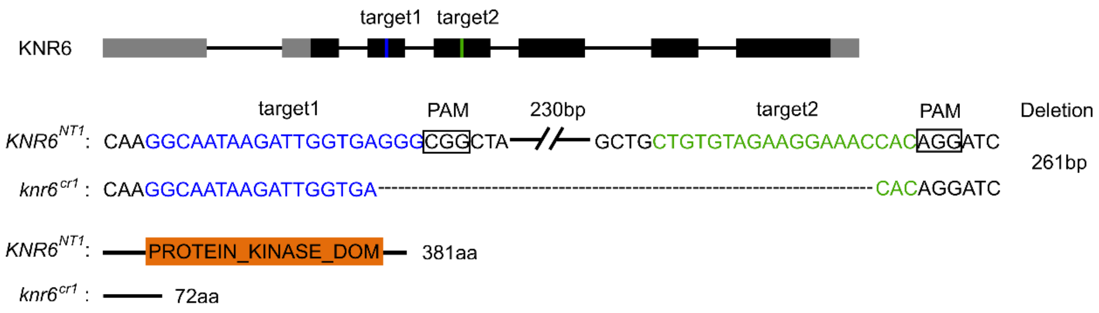

Figure S1 The guide RNAs of KNR6 and editing types in KNR6 knockout lines

The two target sites of gRNAs are marked in blue and green. The protospacer-adjacent motif is boxed, and deletions are indicated by dashes. The two gRNA sites are located in the second and third exons of *KNR6*. The 261-bp deletion and 1-bp insertion led to premature transcriptional termination of KNR6 at the 72nd amino acid residual (aa) and the lack of a protein kinase domain at the 80th amino acid residual, respectively.

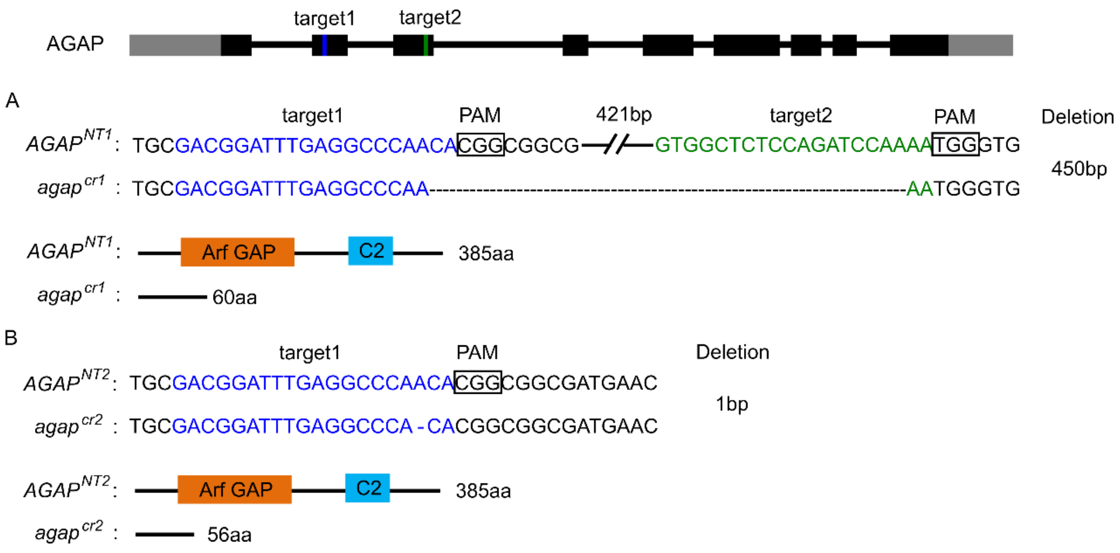

Figure S2 Guide RNAs of AGAP and editing types in AGAP knockout lines

The target site of the gRNA is marked in blue. The protospacer-adjacent motif is boxed, and deletions are indicated by dashes. The gRNAs site is located in the second exon of *AGAP*. The 1-bp deletion led to the premature transcriptional termination of AGAP at the 56th amino acid residual.

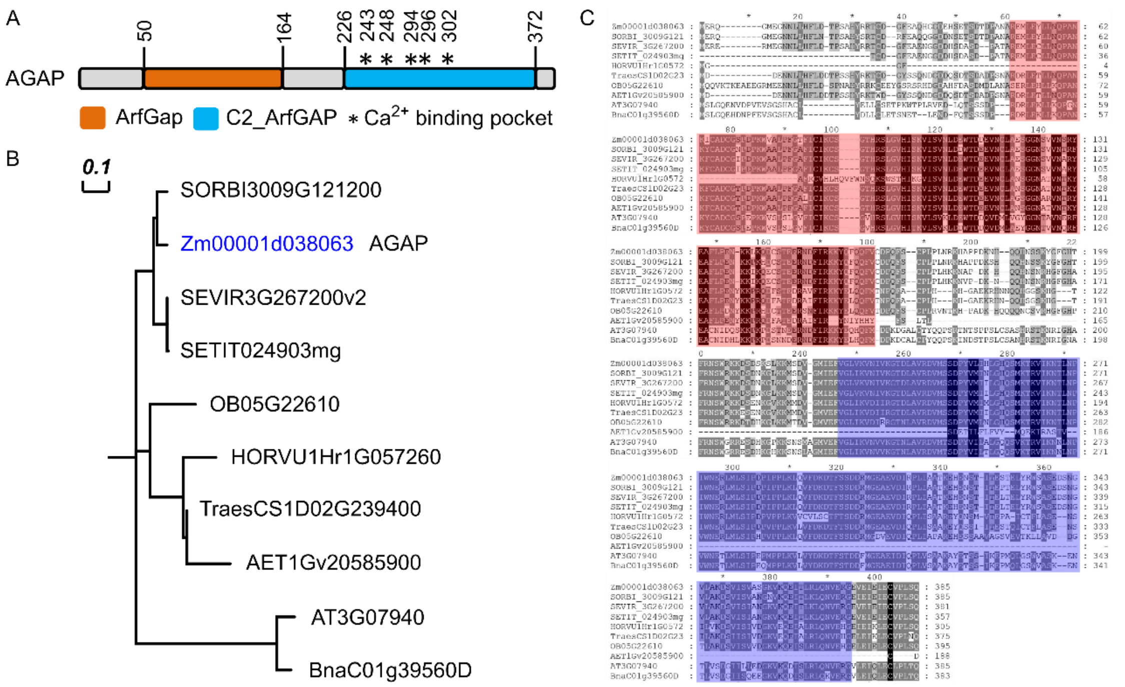

Figure S3 Protein structure, phylogenetic tree and conserved domain analyses of AGAP

AGD (marked in red) and C2_AGAP (marked in blue) domains were conserved across grass species.

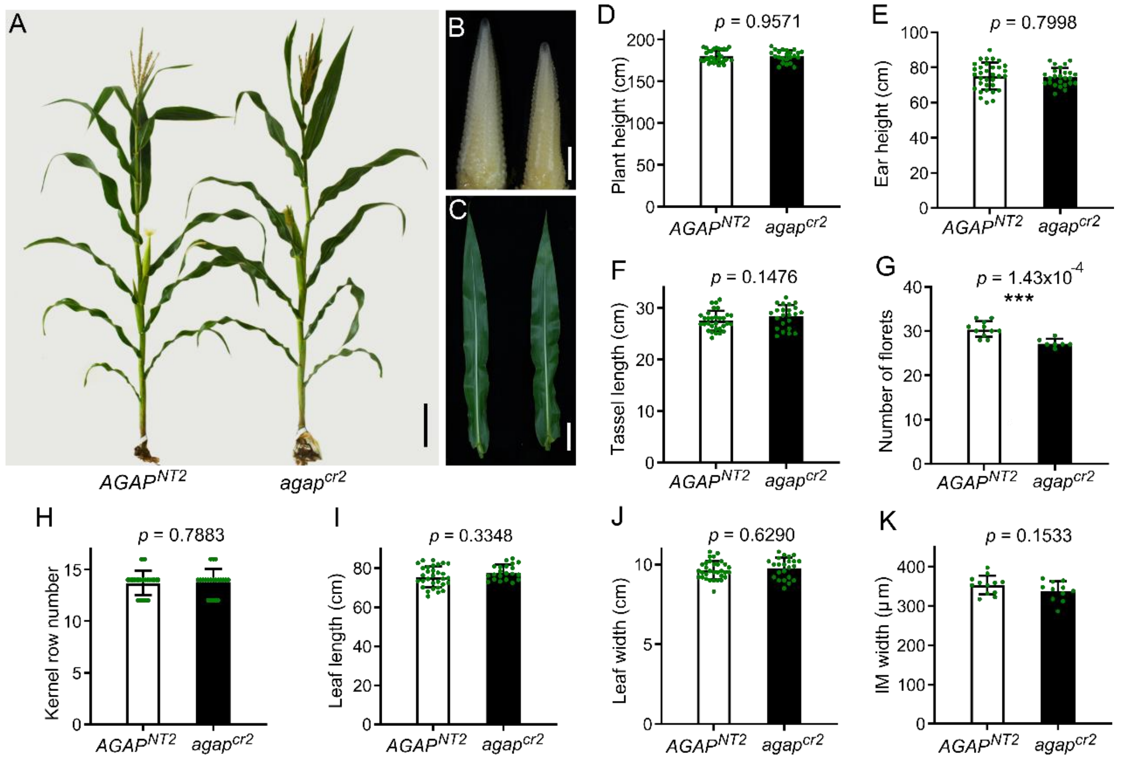

Figure S4 Vegetative and reproductive traits of *AGAP*^{NT2} and *agap*^{cr2}

(**A–C**) States, developing ear inflorescences and leaves of *AGAP*^{NT2} and *agap*^{cr2} plants. Scale bar = 10 cm in (A, C) and 1 mm in (B).

(**D–K**) Statistical results for plant height (D), ear height (E), tassel length (F), number of florets (G), kernel row number (H), leaf length (I), leaf width (J) and inflorescence meristem width (K) between *AGAP*^{NT2} and *agap*^{cr2} plants. The values in (D–K) are the means ± s.d.s, and the significance levels of differences were estimated using a one-way ANOVA. *** indicates a statistical difference at the $p < 0.001$ level.

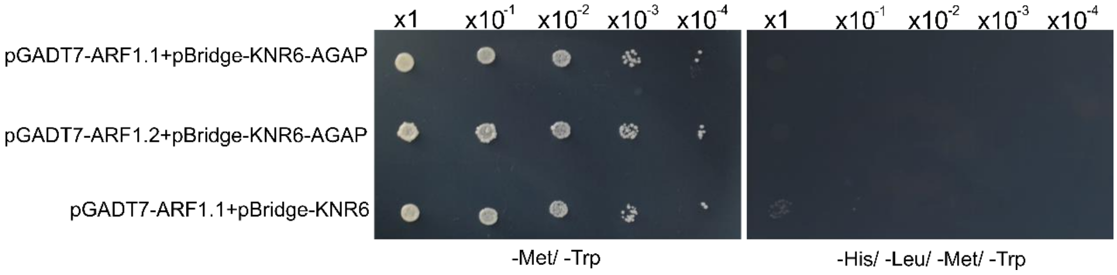

Figure S5 KNR6, AGAP and ARF1 could not form a ternary protein complex

The yeast strains contain KNR6, AGAP and ARFs were diluted (10^{-1}, 10^{-2}, 10^{-3}, 10^{-4}) and grown on SD /-Met/ -Trp and SD /-His /-Leu /-Met/ -Trp plates. The pGADT7-ARF1.1 and pBridge-KNR6 were as negative control.
